# Folliculin Interacts with Rab35 to Regulate EGF-Induced EGFR Degradation

**DOI:** 10.3389/fphar.2017.00688

**Published:** 2017-09-26

**Authors:** Jianchao Zheng, Biao Duan, Shixiu Sun, Jie Cui, Jun Du, Yujie Zhang

**Affiliations:** ^1^Department of Physiology, Nanjing Medical University, Nanjing, China; ^2^Jiangsu Key Lab of Cancer Biomarkers, Prevention and Treatment, Collaborative Innovation Center for Cancer Personalized Medicine, Nanjing Medical University, Nanjing, China; ^3^Department of Biochemistry and Molecular Biology, Nanjing Medical University, Nanjing, China

**Keywords:** EGFR, degradation, FLCN, Rab35, feedback

## Abstract

**Aims and Hypothesis:** This study aims to investigate the mechanism involved in intracellular regulation of EGFR degradation induced by EGF.

**Methods:** Phosphorylation of proteins related to EGFR signaling was examined by western blot analysis. Activation, connection between Rab35 and folliculin (FLCN) were assessed by pulldown, coimmunoprecipitation assays separately. The relationship between FLCN and cell growth was detected using gene overexpression and knock-down techniques.

**Results:** Here, we demonstrate that interfering with FLCN, a tumor suppressor, reduces the rate of EGF-induced EGFR degradation, resulting in prolonged activation of downstream signaling. Rab35 is also involved in these processes. Moreover, C-terminal of FLCN binds to and activates Rab35. Of special interest is the observation that erlotinib, a selective EGFR inhibitor, not only obstructs the EGFR-mediated cellular signaling, but also abolishes EGF-stimulated EGFR degradation. Further results reveal that EGF facilitates the activation of Rab35, and FLCN modulates EGF-dependent Rab35 activation and cell growth.

**Conclusions:** Taken together, our study proposes a negative-feedback regulation model in which FLCN mediates EGF-induced Rab35 activation, thereby increasing EGFR degradation and attenuating EGFR signaling.

## Introduction

Epidermal growth factor receptor (EGFR), the first receptor tyrosine kinase (RTK) discovered more than three decades ago, has emerged as a central regulator of critical cellular processes including proliferation, differentiation and motility. EGFR achieves these functions mainly by activating RAF-MEK-ERK1/2 and PI3K-AKT signaling cascades (Ullrich et al., 1984; Hunter, 2000; Schlessinger, 2000). Excessive activation of EGFR and of the downstream signal transducers are found in many diverse cancers. Therefore, drugs that target the EGFR signaling pathway have been approved for the treatment of cancer (Tebbutt et al., 2013; Tomas et al., 2014). Unfortunately, an increasing number of studies revealed that a complex network of feedback regulation triggers the resistance to these target therapies and limits their overall clinical benefits (Avraham and Yarden, 2011; Lee et al., 2014; Li and Mansmann, 2014).

Receptor endocytosis is a major negative feedback loop that plays a key role in spatiotemporal regulation of signaling (Avraham and Yarden, 2011; Er et al., 2013; Jones and Rappoport, 2014). Binding of ligands to EGFR induces EGFR autophosphorylation at the plasma membrane and its internalization to the early endosome, where EGFR is sorted for recycling to the cell surface or degradation in the lysosome. The fate of the EGFR is influenced by cellular context, ligand type, concentration and duration of stimulation, and has significant consequences for cellular signaling outputs (Tomas et al., 2014). Although some reports showed that the inability of EGFR internalization and degradation is implicated in carcinogenesis, the mechanisms related to carcinogenesis remain incomplete (Deribe et al., 2009; Goueli et al., 2012).

Rab GTPases constitute the largest family of small GTPases. Together with their regulators, they control many aspects of intracellular trafficking. Rab GTPases cycle between active GTP-bound and inactive GDP-bound states by GEF, GAP, and other GTPase-activating proteins (Stenmark, 2009). Rab35, a highly conserved Rab during evolution, predominantly functions in endosomal trafficking by controlling the location of cargo such as transferrin, β1-integrin, cadherin (Patino-Lopez et al., 2008; Allaire et al., 2013; Charrasse et al., 2013; Argenzio et al., 2014; Tang et al., 2015). Aberrant Rab35 expression and gene mutation are associated with malignance, however, the oncogenic as well as tumor suppressive function of Rab35 have been reported previously (Sheach et al., 2009; Allaire et al., 2013; Zhu et al., 2013; Tang et al., 2015; Wheeler et al., 2015). In particular, Rab35 knockdown significantly enhances EGFR signaling and cell migratory capacity (Allaire et al., 2013; Wheeler et al., 2015). However, whether and how Rab35 contributes to EGFR trafficking remains largely unknown.

Folliculin (FLCN), a tumor suppressor, was originally identified from patients with Birt–Hogg–Dubé syndrome (Nickerson et al., 2002; Schmidt and Linehan, 2015). Subsequent studies demonstrated that it is mutated in various types of cancers including endometrial carcinoma, gastric cancer and colorectal cancer (Fujii et al., 2006; Jiang et al., 2007; Guda et al., 2015). Mechanically, FLCN deficiency in mice facilitates the activation of RAF, MEK, ERK1/2 and AKT (Baba et al., 2008; Hasumi et al., 2009), which are the key transducers of EGFR signaling. Interestingly, crystallographic and biochemical analysis revealed that DENN domain of FLCN has GEF activity toward Rab35 (Nookala et al., 2012). In this study, we hypothesize that FLCN may function as an activator of Rab35 and regulate EGFR intracellular trafficking and its downstream signaling transduction. Here, we show the C-terminal of FLCN binds to Rab35. We moreover establish a negative-feedback regulation model in which FLCN mediates EGF-induced Rab35 activation, thereby increasing EGFR degradation and attenuating EGFR signaling.

## Materials and methods

### Cell lines and cell culture

Human ovarian cancer cell line HeLa and human embryonic kidney cell line HEK293T were obtained from the Cell Biology Institute of Chinese Academy of Sciences (Shanghai, China). All cells were cultured in Dulbecco's modified Eagle's medium (DMEM, high glucose; Gibco, Thermo Scientific, Grand Island, NY, USA) supplemented with 10% (v/v) fetal bovine serum (FBS; Gibco) and antibiotics (100 U/mL streptomycin and 100 μg/mL penicillin; Invitrogen, USA) in a humidified incubator at 37°C with 5% CO_2_.

### Plasmids and siRNAs

The full-length FLCN plasmid was kindly provided by Dr. Laura S. Schmidt (Department of Urologic Oncology, NCI, USA). Full-length FLCN cDNA was amplified from this plasmid using the following primer set, sense: 5′-CCCAAGCTTATGAATGCCATCGTGGCTC-3′ and antisence: 5′-GCTCTAGATCAGTTCCGAGACTCCGAG-3′. The polymerase chain reaction (PCR) product was cloned into the pCMV-N-Flag (Beyotime, Nantong, China). FLCN cDNA fragments corresponding to the N-terminal (FLCN-NT), C-terminal (FLCN-CT) of FLCN were amplified using the following primer set, sense: 5′-CCCAAGCTTCGGAAGCTGCCAGTCTTC-3′ and antisence: 5′-GCTCTAGATCAGGGCTGCCAGCTCCCACA-3′ and sense: 5′-CCGGAATTCC GGAAGCTGCCAGTCTTC-3′ and antisence: 5′-CCGCTCGAGTCAGTTCCGAGACTCCGAG−3′ by PCR and ligated into pCMV-N-Flag. pEGFP-Rab35 (WT, S22N, and Q67L) plasmids were kindly provided by Dr. Matthew P. Scott (Department of Medicine, Stanford University, Stanford, California, USA). Full-length Rab35 cDNA was amplified from the Rab35 WT plasmid using the following primer set, sense: 5′-CGGAATTCATGGCCCGGGACTACGAC-3′ and antisence: 5′-CCGCTCGAGTTAGCAGCAGCGTTTCTT-3′. The PCR product was cloned into the pGEX-4T-1. The cells were seeded in six-well plate, cultured to 80% confluence, and then transfected with those plasmids by ExFect™ Transfection Reagent (Vazyme Biotech, Piscataway, NJ, China).

The small interfering RNA (siRNA) were chemically synthesized and purified by China GenePharma Co. FLCN: 5′-GAUAAAGAGACCUCCAUUAdTdT-3′; and Rab35: #1, 5′-GCAGCAACAACAGAACGAUdTdT-3′, #2, 5′-GCUCACGAAGAACAGUAAAdTdT-3′ and #3, 5′-GAUGAUGUGUGCCGAAUAUdTdT-3′. Cells were transfected with siRNA by Lipofectamine 2000 (Invitrogen).

### Reagents and antibodies

EGF was purchased from R&D systems (Minneapolis, MN, USA). Cyclohexamide (CHX) and 3-(4,5-dimethylthiazol-2-yl)-2,5-diphenyltetrazolium bromide (MTT) were purchased from Sigma (USA). Erlotinib was purchased from ApexBio Technology (USA). Phospho-ERK1/2, Phospho-AKT (473), AKT, GFP, FLCN rabbit antibodies, and ERK1/2 mouse antibodies were purchased from Cell Signaling Technology (Danvers, MA, USA). Normal mouse IgG, normal rabbit IgG, GAPDH and EGFR rabbit antibodies were purchased from Santa Cruz Biotechnology (Santa Cruz, CA, USA). GFP mouse antibody was purchased from EarthOx (USA). Rab35 rabbit antibody was purchased from Biogot technology (Nanjing, Jiangsu, China). Flag mouse antibody was purchased from Sigma. HRP-conjugated secondary antibody was purchased from Santa Cruz Biotechnology.

### Western blot

Sample protein extraction and concentration determination of whole cells were performed as previously described (Deng et al., 2016). Briefly, equal amounts of protein were run on SDS polyacrylamide gels and transferred to nitrocellulose membrane. The resulting blots were blocked with 5% non-fat dry milk and incubated with primary antibodies overnight at 4°C. Protein bands were then detected by incubating with HRP-conjugated secondary antibodies for 2 h at room-temperature and visualized with ECL reagent (Millipore, Billerica, MA, USA) by Chemi Doc XRS and gel imaging system (Bio-Rad, USA). Densitometry analysis was performed using Quantity One software and band intensities were normalized to those of GAPDH.

### *In vitro* pull down assays and immunoprecipitation

All cells were lysed with lysis buffer (20 mM Tris PH7.5, 150 mM NaCl, 1% Triton X-100, 2.5 mM sodium pyrophosphate, 1 mM EDTA, 1% Na_3_VO_4_, 0.5 μg/ml leupeptin, 1 mM PMSF). For *in vitro* binding assays, GST fusion proteins were first purified on MagneGST glutathione particles (Promega, Madison, WI). Five hundred micrograms of cell lysates transfected with target proteins were then incubated with GST fusion proteins. For immunoprecipitation assays, 500 μg of cellular proteins were precipitated and ralated protein were detected by western blot, as described previously (Duan et al., 2016). Antibodies against FLCN, Rab35 and GFP were used at dilutions of 1:100, 1:50, and 1:100 for immunoprecipitation, respectively.

### Measurement of Rab35 activity

The Rab35-binding domain (RBD35) of mRUSC2 (aa 739–862) was amplified by PCR and ligated into pGEX-2T using EcoRI and SalI sites. It was generated as previously reported. GST-RBD35 was expressed in bacteria and purified by MagneGST glutathione particles at 4°C with constant rotation for 30 min. HeLa cells were serum-starved for 12 h and treated with 10 ng/mL EGF (5–60 min), then proteins were extracted and activation of Rab35 was measured as previously described (Deng et al., 2016).

### MTT assay

Cells transfected with plasmids or siRNA were seeded at a density of 1 × 10^5^ cells per well into 96-well plate and treated with EGF for the indicated times and doses. After culture, cells were washed, MTT was added and the plate was incubated in the dark for 4 h, followed by measurement of absorbance value at 490 nm using a microplate absorbance reader (Bio-Tek, Elx800, USA). The fold growth was calculated as the absorbance of drug treated sample/control sample absorbance × 100%.

### EdU staining

Cell proliferation was measured using EdU staining kit according to the manufacturer's instruction (RiboBio, Guangzhou, China). In brief, cells were cultured in 96-well plate until reaching 70% confluence, then EdU was added to the culture media for 2 h. After fixing by paraformaldehyde, the cells were washed with PBS. Then, the cells were incubated with glycine and washed with PBS containing 0.5% Triton X-100. After the cells were counterstained with hochest33342, cells were mounted and imaged by fluorescence microscopy.

### Statistical analysis

Data were presented as mean ± standard error of the mean (*SD*). Statistical analyses were performed using Prism 5.0 software (GraphPad Software, USA). Student's *t*-test was used for comparison between groups. Values of *P* < 0.05 were considered statistically significant. All experiments were repeated at least three times.

## Results

### Active Rab35 promotes EGFR degradation and attenuates EGFR signaling

**Figure 1:**
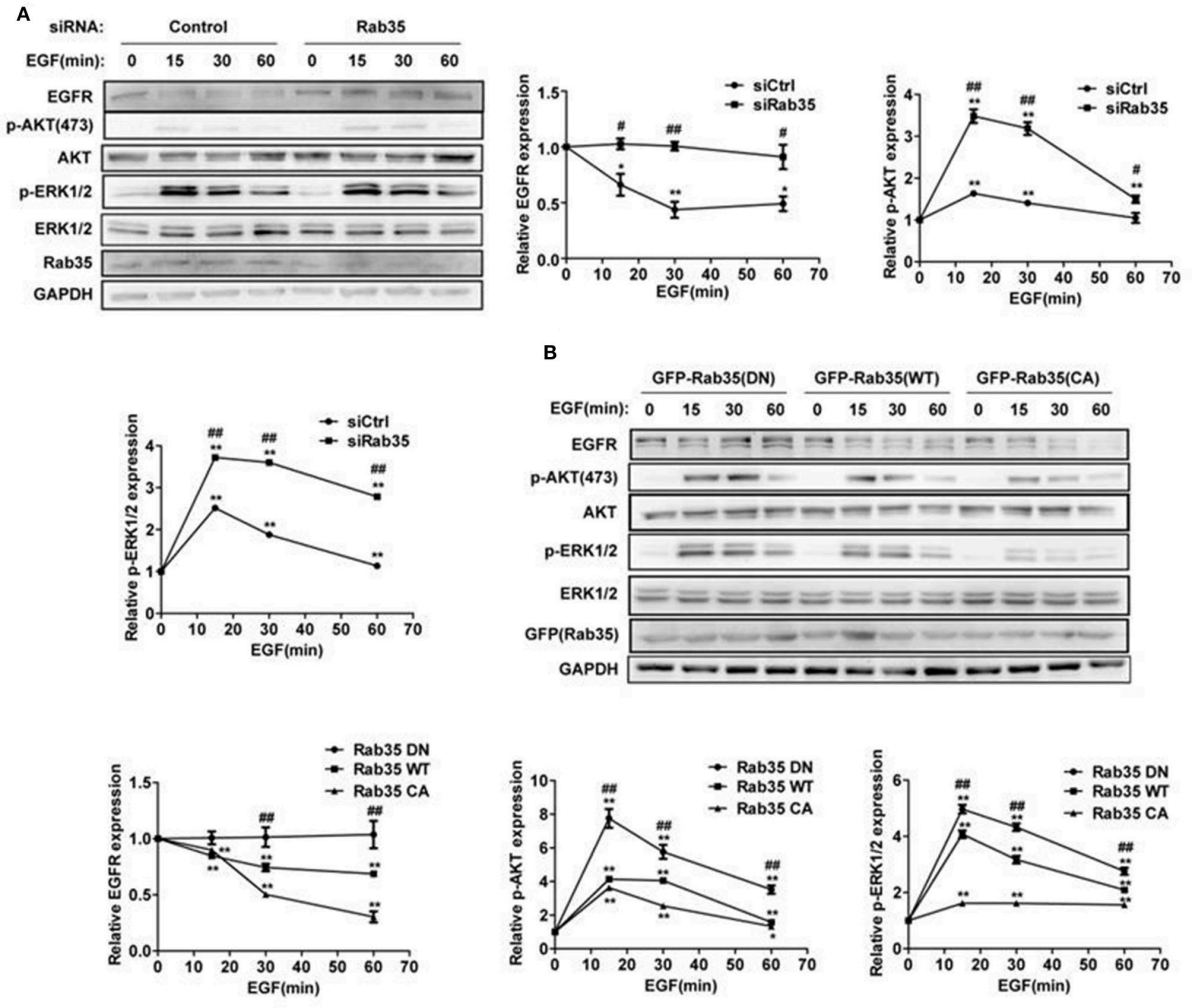
Rab35 activation promotes EGFR degradation and attenuates EGFR signaling. **(A)** HeLa cells were starved overnight and then treated with cycloheximide for 30 min, then stimulated with 10 ng/mL EGF for the indicated time. EGFR, p-Akt and p-ERK1/2 levels were detected by western blot. Data were presented as mean ± *SD* of 3 independent determinations. **(B)** Overexpression of different plasmids of Rab35 (Rab35 DN, Rab35 WT, and Rab35 CA) to detect EGF-induced EGFR degradation and its downstream signaling. ^*^P < 0.05 and ^**^P < 0.01 show the comparison between groups at the different time points of EGF stimulation. ^#^*P* < 0.05 and ^##^*P* < 0.01 show the comparison between different groups at a same time point.

A previous study showed that knock down of Rab35 significantly enhanced the serum-induced EGFR recycling and signaling in both COS-7 and U251 cells (Allaire et al., 2013). In order to address whether Rab35 regulates EGFR abundance and signaling with EGF stimulation, we assessed EGFR degradation in HeLa cells, a model cell line with moderate EGFR abundance and complete endomembrane system (Sigismund et al., 2005; Spangler et al., 2010). Serum-starved HeLa cells were stimulated with EGF (10 ng/mL) for indicated times in the presence of the protein synthesis inhibitor cycloheximide (CHX). We observed EGF-induced degradation of EGFR, and this model was used in the following experiments. Compared with control cells, cells depleted of Rab35 by siRNA suppressed EGF-induced EGFR degradation and enhanced EGFR signaling (Figure 1A). To confirm these results, we repeated EGFR degradation assays by overexpression of three different constructs of Rab35 in HeLa cells. Dominant negative GDP-locked Rab35-S22N (Rab35 DN) consistently delayed EGFR degradation and enhanced the activation of ERK1/2 and AKT compared with wild-type Rab35 (Rab35 WT), while constitutively active GTP-locked Rab35-Q67L (Rab35 CA) facilitated EGFR degradation and attenuated EGFR signaling (Figure 1B). Together, these data suggest that active Rab35 is necessary to promote EGF-induced EGFR degradation and reduce EGFR signaling activation.

### FLCN regulates EGFR degradation and its downstream signaling

**Figure 2:**
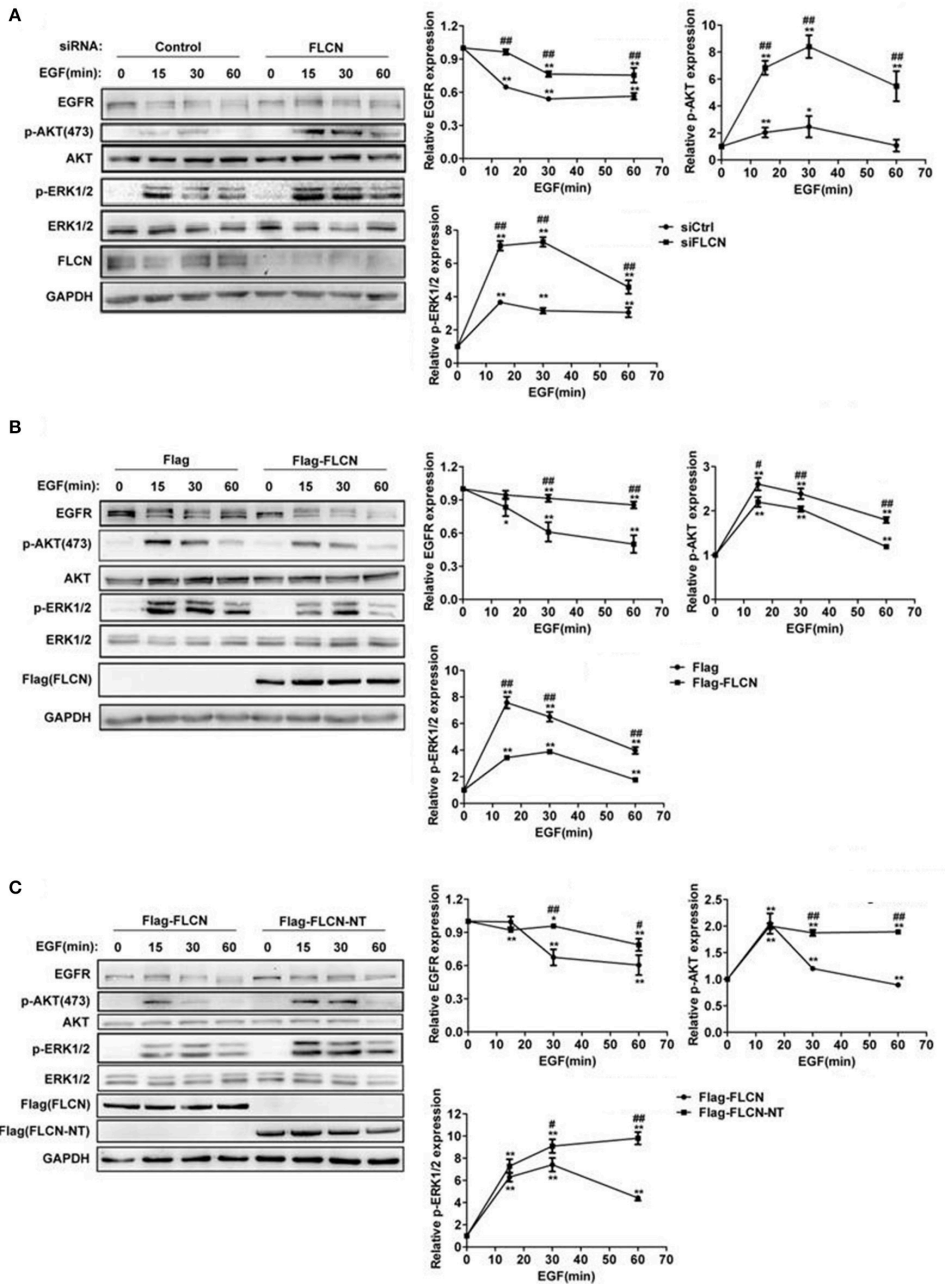
FLCN promotes EGFR degradation and attenuates EGFR signaling. **(A)** HeLa cells were transfected with negative control siRNA or siRNA pool for FLCN, **(B)** pCMV-Flag empty vector or Flag-FLCN, **(C)** Flag-FLCN, or FLCN-NT. Cells were lysed and subjected to western blot analysis for EGFR expression and phosphorylation levels of its downstream signaling. ^*^P < 0.05 and ^**^P < 0.01 show the comparison between groups with EGF stimulation and without EGF stimulation. ^#^*P* < 0.05 and ^##^*P* < 0.01 show the comparison between different groups at a same time point.

Considering FLCN has GEF activity toward Rab35 (Nookala et al., 2012), we hypothesized that FLCN might also affect the degradation of EGF-induced EGFR and its downstream signaling pathways. To test this, we used EGF to stimulate HeLa cells after transfected with FLCN siRNA pool. The results showed that EGF-stimulated EGFR degradation was significantly slowed down in FLCN-silencing HeLa cells, and knockdown of FLCN resulted in persisting activation of p-AKT and p-ERK (Figure 2A). Moreover, when FLCN is overexpressed, the EGF-induced EGFR degradation was accelerated and the expressions of p-AKT, p-ERK were significantly weakened (Figure 2B).

It has been reported that FLCN deficit was mostly due to BHD gene mutation in exon 11, resulting in a C-terminal deletion mutant of FLCN (Schmidt et al., 2005; Hasumi et al., 2009). To prove whether this mutant could affect EGFR degradation and its downstream signaling, we transiently transfected HeLa cells with FLCN full length (FLCN) and N-terminal (FLCN-NT), which is a truncated FLCN analogous to the BHD gene mutation to validate the function of FLCN (Nookala et al., 2012). The result showed that compared with the Flag-FLCN-NT, overexpression of full-length FLCN accelerates EGFR degradation and decreased p-AKT and p-ERK levels (Figure 2C). These results indicated that C-terminal deletion mutant of FLCN could not facilitate EGFR degradation and attenuate its downstream signaling.

### FLCN binds to Rab35

**Figure 3:**
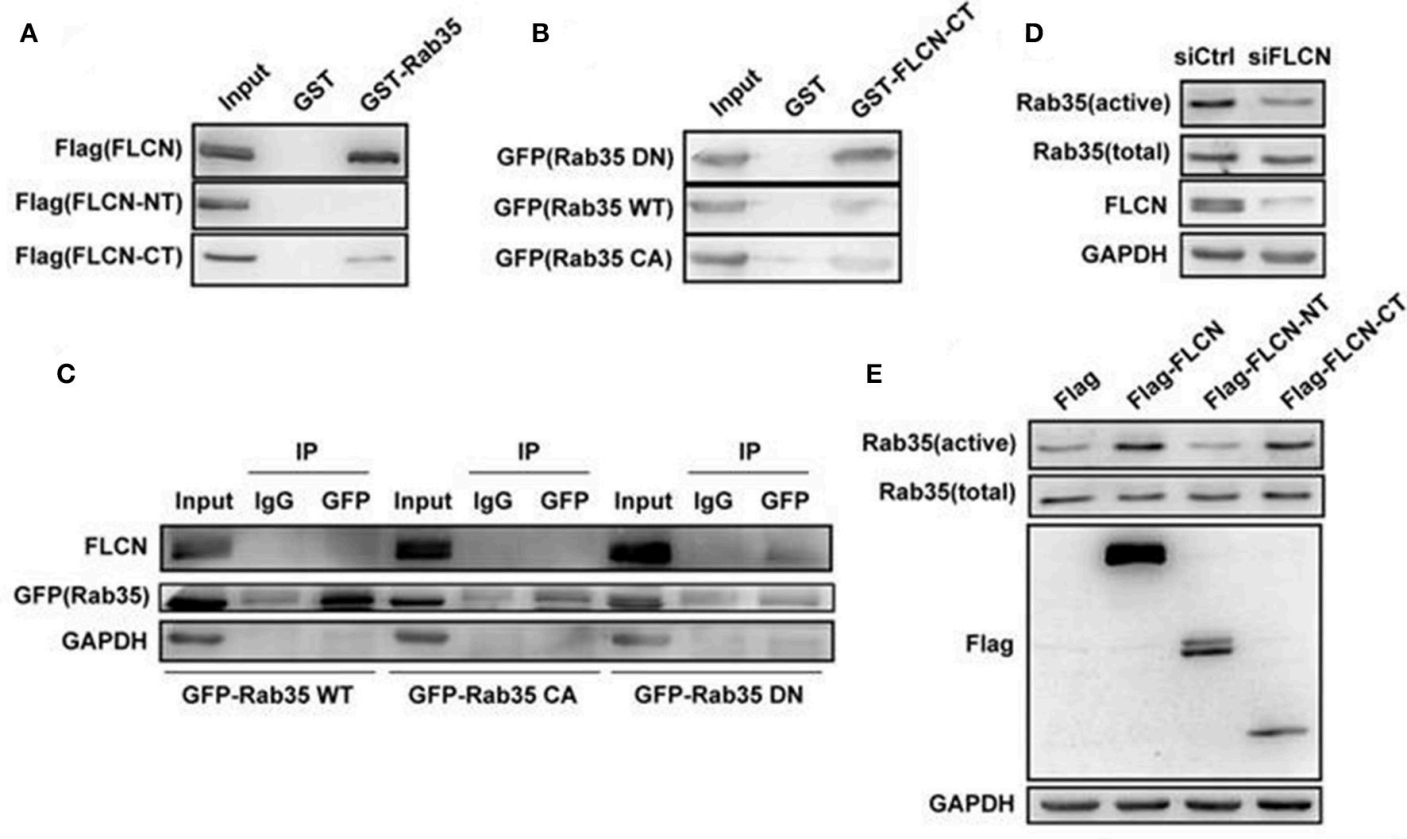
FLCN functions as an activator toward Rab35 *in vitro*. **(A)** HEK293T cells were transiently transfected with Flag-FLCN, Flag-FLCN-NT, or Flag-FLCN-CT plasmid. At 36 h after transfection, lysates of HEK-293T cells was incubated *in vitro* with GST-tagged Rab35 or GST alone, and coprecipitation of FLCN with GST fusion Rab35 proteins, bound to glutathione-beads, was analyzed by western blot using an anti-Flag antibody. GST, GST alone used as a control. **(B)** HEK293T cells were transiently transfected with Rab35 CA, Rab35 WT, or Rab35 DN plasmid. At 36 h after transfection, lysates of HEK-293T cells was incubated *in vitro* with GST-tagged FLCN-CT or GST alone, and coprecipitation of Rab35 with GST fusion FLCN-CT proteins, bound to glutathione-beads, was analyzed by western blot using an anti-GFP antibody. GST, GST alone used as a control. **(C)** Equal amounts of lysates from the indicated HeLa cells transiently transfected with Rab35 CA, Rab35 WT, or Rab35 DN plasmid were immunoprecipitated with an anti-GFP antibody or IgG, and then both unprocessed lysates (Input) and immunoprecipitates were resolved by western blot using the indicated antibodies. **(D)** HEK293T cells were transiently transfected with negative control siRNA or FLCN siRNA pool. At 36 h after transfection, cells were lysed and subjected to GST-pulldown analysis for the activity of Rab35. **(E)** HEK293T cells were transiently transfected with pCMV-Flag empty vector, Flag-FLCN, Flag-FLCN-NT, or Flag-FLCN-CT plasmid. At 36 h after transfection, cells were lysed and subjected to GST-pulldown analysis for the activity of Rab35.

In order to further study the relationship between FLCN and Rab35, we overexpressed FLCN, FLCN-NT, FLCN-CT in HEK293T cells and performed GST-pulldown assays to detect the combination between Rab35 WT and different fragments of FLCN. Results showed that GST-Rab35 bound preferentially to full-length of FLCN, followed by FLCN-CT but not FLCN-NT (Figure 3A). To further validate the relationship of FLCN to Rab35, Rab35DN, and Rab35 WT plasmids were overexpressed in HEK293T cells, respectively. We found that FLCN-CT bound preferentially to Rab35 DN, rather than Rab35 WT or Rab35 CA (Figure 3B), which supports the idea that FLCN is a Rab35 GEF in cells. By immune coprecipitation, we demonstrated that endogenous FLCN binds to Rab35 DN, but not Rab35 WT or Rab35 CA (Figure 3C). Moreover, silencing of FLCN decreased Rab35 activity significantly (Figure 3D). We also found that overexpression of FLCN and FLCN-CT, but not FLCN-NT, significantly enhanced Rab35 activation (Figure 3E). Together, these results indicate that FLCN might act as GEF of Rab35 and its C-terminal domain might be required for its GEF activity.

### EGF activates Rab35

**Figure 4:**
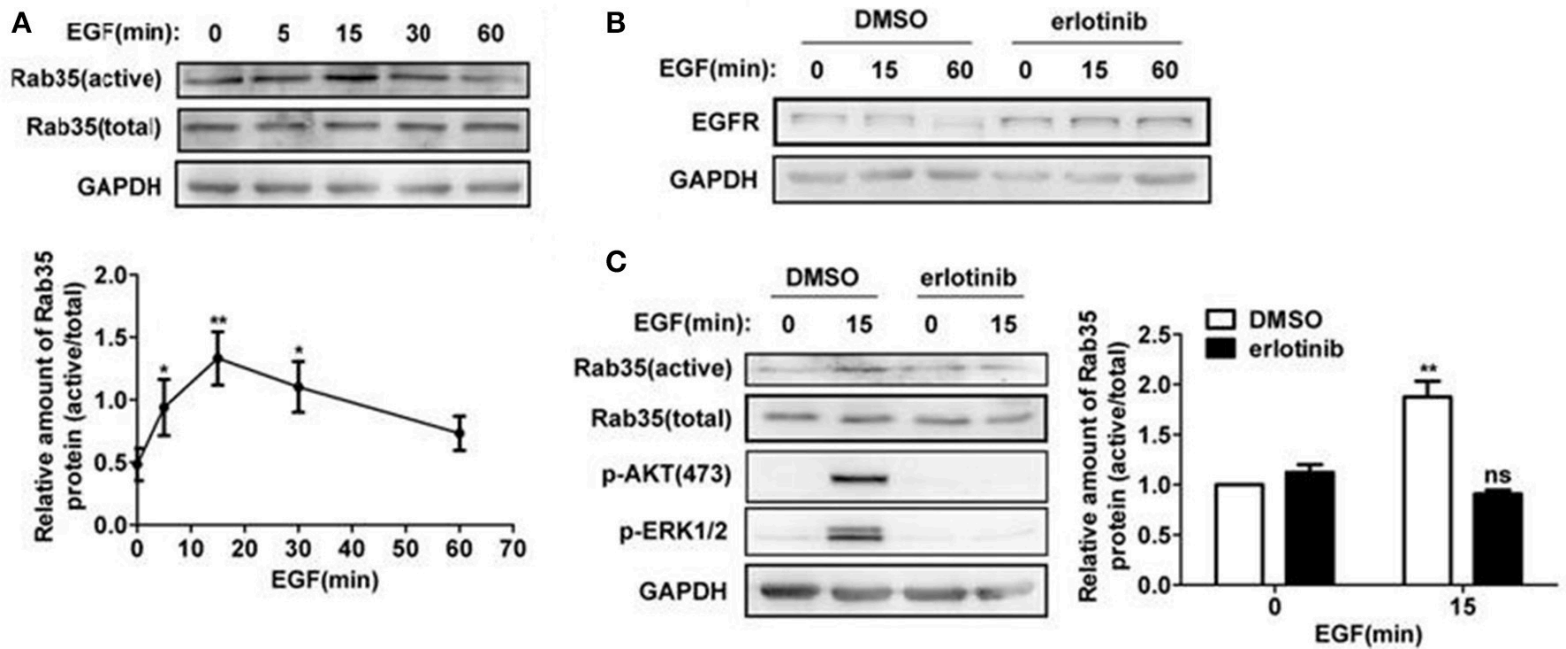
EGF activates Rab35 via EGFR. **(A)** HeLa cells were serum-starved for 12 h and stimulated with 10 ng/ml EGF for the indicated time. The GTP-bound form of endogenous Rab35 was precipitated by GST-RBD35 through GST-pulldown analysis. **(B)** HeLa cells were serum-starved and treated with DMSO or 1 μM erlotinib overnight, and then stimulated with 10 ng/ml EGF for the indicated time. Cells were lysed and subjected to western blot analysis for EGFR expression. ^*^P < 0.05 and ^**^P < 0.01 show the comparison between groups with EGF stimulation and without EGF stimulation. **(C)** HeLa cells were serum-starved and treated with DMSO or 1 μM erlotinib overnight, and then stimulated with 10 ng/ml EGF for 15 min or not. Cells were lysed and subjected to GST-pulldown analysis for the activity of Rab35. ^**^P < 0.01 show the comparison between groups with EGF stimulation and without EGF stimulation, ns (not significant).

To study whether EGF activates Rab35, we stimulated HeLa cells with EGF for 0, 5, 15, 30, and 60 min and then detected Rab35 activity by GST-pulldown assays. As shown in Figure 4A, Rab35 activity reached a peak when EGF stimulated cells for 15 min. To further confirm the role of EGF-EGFR to Rab35, erlotinib was used to inhibit EGFR activity. The results showed that erlotinib inhibited EGFR degradation upon EGF stimulation (Figure 4B). This indicates that EGFR degradation depends on the activation of EGFR. Further experimental results show that erlotinib pretreatment also prevented EGF-induced Rab35 activity in HeLa cells (Figure 4C) as well as in the gastric cancer cell line SGC-7901 (data not shown).

### FLCN mediates EGF-induced Rab35 activation

**Figure 5:**
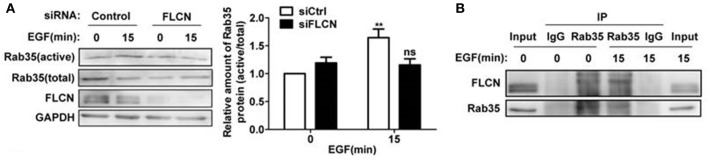
FLCN mediates EGF-induced Rab35 activation. **(A)** HeLa cells were transiently transfected with negative control siRNA or FLCN siRNA pool. At 36 h after transfection, cells were serum-starved overnight and stimulated with 10 ng/ml EGF for 15 min. Cells were lysed and subjected to GST-pulldown analysis for the activity of Rab35. ^**^P < 0.01 show the comparison between groups with EGF stimulation and without EGF stimulation, ns (not significant). **(B)** HeLa cells were serum-free overnight and then treated with 10 ng/ml EGF for 15 min. Cells were lysed and anti-Rab35 or IgG immunoprecipitates were analyzed for coprecipitation of FLCN by western blot. Unprocessed lysates of HeLa cells was used as input.

In the above studies, we found that FLCN binds to Rab35, but whether FLCN can regulate Rab35 activity in the process of EGF-induced Rab35 activation is unknown. The results here showed that the increased Rab35 activity by EGF stimulation was attenuated when FLCN was silenced in HeLa cells (Figure 5A), suggesting that FLCN mediates EGF-stimulated activation of Rab35. To investigate the role of EGF in regulating the interaction between FLCN and Rab35, we detected the interaction status of FLCN and Rab35 by EGF stimulation. We found that the amount of endogenous Rab35 coprecipitated with FLCN were increased upon EGF stimulation for 15 min (Figure 5B).

### Rab35 acts as a downstream effector to control EGFR degradation

**Figure 6:**
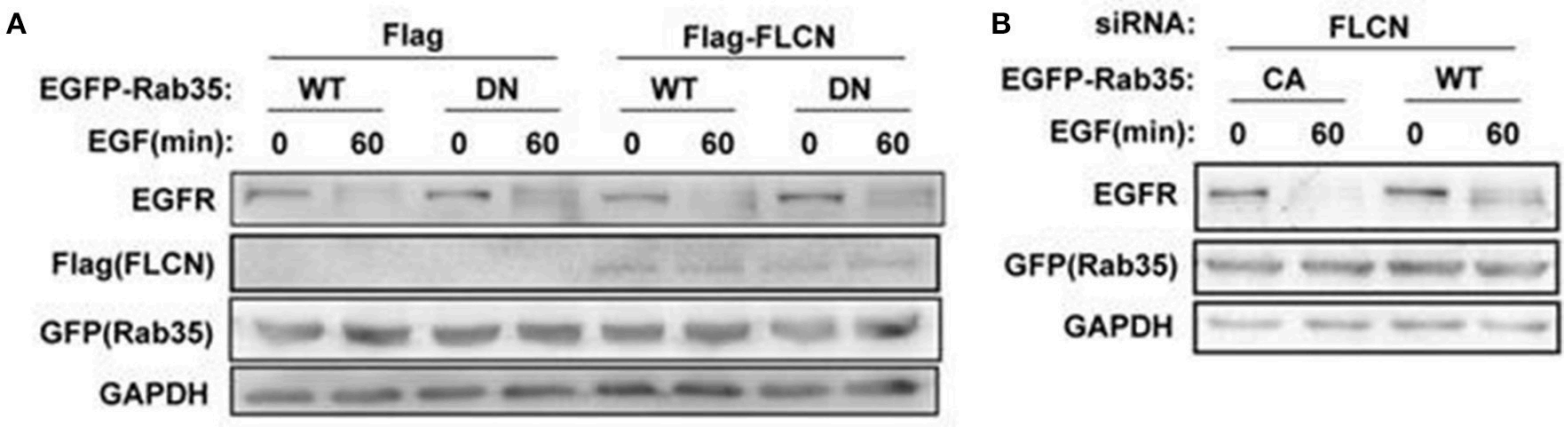
EGFR degradation requires Rab35 activation. **(A)** HeLa cells were transiently transfected with Rab35 WT or Rab35 DN plasmid and pCMV-Flag empty vector or Flag-FLCN at the same time. Cells were lysed and subjected to western blot analysis for EGFR expression. **(B)** FLCN-silenced HeLa cells were transiently transfected with Rab35 CA or Rab35 DN plasmid. Cells were lysed and subjected to western blot analysis for EGFR expression.

To investigate whether FLCN regulates EGFR degradation through Rab35, Rab35 CA or Rab35 DN were transfected in FLCN-silencing or FLCN-overexpression cells to detect EGF-induced EGFR degradation separately. The results show that after transfection with Rab35 DN, the degradation of EGFR in HeLa cells was slowed down, whether Flag-FLCN existed in cells or not (Figure 6A). Interestingly, overexpression of Rab35 CA accelerates EGFR degradation when FLCN was interfered with siRNA (Figure 6B). Together, these results indicate that Rab35 may promote EGF-induced EGFR degradation via FLCN-independent manner.

### FLCN decreases EGF-induced cell growth

**Figure 7:**
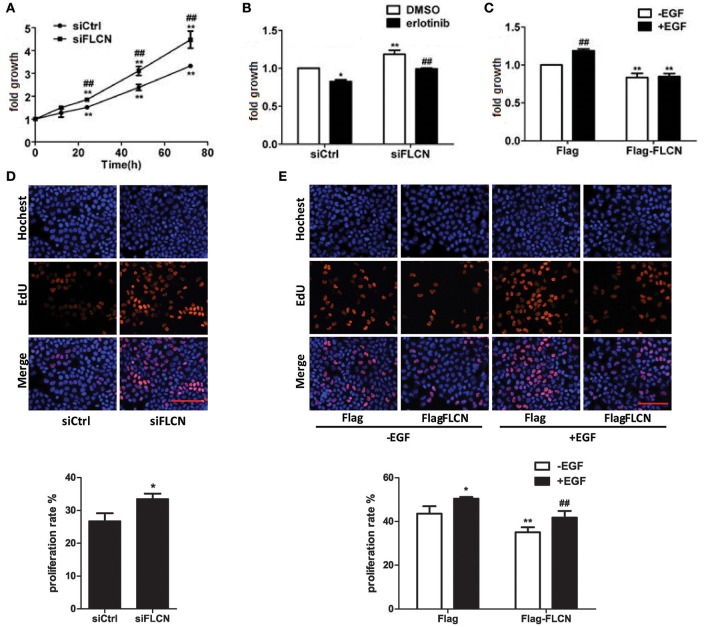
FLCN involves in EGF-induced cell growth. **(A)** HeLa cells were transiently transfected with negative control siRNA or FLCN siRNA pool. At the indicated time points after transfection, cells were analyzed by MTT assay. ^*^P < 0.05 and ^**^P < 0.01 show the comparison between the different time points and the 0 h point in the same group. ^#^*P* < 0.05 and ^##^*P* < 0.01 show the comparison between different groups at a same time point. **(B)** HeLa cells were transiently transfected with negative control siRNA or FLCN siRNA pool and treated with DMSO or 1 μM erlotinib for 72 h, then cells were analyzed by MTT assay. ^*^P < 0.05 and ^**^P < 0.01 show the comparison with the group transfected with negative control siRNA and treated with DMSO. ^##^*P* < 0.01 show the comparison between groups treated with DMSO and 1 μM erlotinib. **(C)** HeLa cells were transiently transfected with pCMV-Flag empty vector or Flag-FLCN plasmid and stimulated with 10 ng/ml EGF for 72 h, then the cells were analyzed by MTT assay. ^**^P < 0.01 show the comparison between groups transfected with pCMV-Flag empty vector and Flag-FLCN plasmid. ^##^*P* < 0.01 show the comparison between groups with EGF stimulation and without EGF stimulation. **(D)** HeLa cells were transiently transfected with negative control siRNA or FLCN siRNA pool for 72 h, then cells were analyzed by EdU staining. ^*^P < 0.05 show the comparison with the group transfected with negative control siRNA and FLCN siRNA. Scale bar, 100 μm. **(E)** HeLa cells were transiently transfected with pCMV-Flag empty vector or Flag-FLCN plasmid and stimulated with 10 ng/ml EGF for 72 h, then the cells were analyzed by EdU staining. ^*^P < 0.05, ^**^P < 0.01 show the comparison with group transfected with pCMV-Flag empty vector with other groups. ^##^*P* < 0.01 show the comparison between EGF-incubation groups transfected with pCMV-Flag empty vector and Flag-FLCN plasmid. Scale bar, 100 μm.

EGFR signaling is a central regulator of cellular processes such as viability and proliferation, According to the results of Peter S. McPherson's lab, knockdown of Rab35 significantly increases proliferation rates of COS-7 cells (Allaire et al., 2013). Then, we investigated the effect of FLCN on cell growth using MTT assay and EdU staining. The results showed that the cell growth rate increased after knockdown of FLCN (Figures 7A,D). However, when FLCN-silencing cells were pretreated with erlotinib, cell growth velocity was decreased (Figure 7B). On the contrary, overexpression of FLCN significantly repressed EGF-induced upregulation of cell growth (Figures 7C,E). Data from Gyorffy et al. also showed that expression of FLCN is positive correlation with patient survival in lung cancer and gastric cancer (Gyorffy et al., 2010). Overall, these results showed that FLCN may affect cell growth in an EGFR dependent manner.

## Discussion

EGFR is shown mutated in a number of tumor types (Lynch et al., 2004; Walker et al., 2009), such mutation leads to its constant activation, which produces uncontrolled cell proliferation. This clinical observation led to the development of anticancer therapeutics directed against EGFR. However, until now, the precise mechanisms about intracellular regulation of EGFR content remain elusive. Similar to EGF (Levi-Montalcini and Cohen, 1960), mice with inactive FLCN in the epidermis have striking delays in eyelid opening (Medvetz et al., 2012), suggesting that FLCN may participate in EGF-EGFR signaling. FLCN has been used to modulate expression and localization of membrane proteins (Medvetz et al., 2012; Nahorski et al., 2012; Goncharova et al., 2014). Here, we propose a model in which negative feedback of FLCN regulates EGFR signaling. FLCN mediates EGF-induced Rab35 activation, leading to EGFR degradation and decreased activity of its downstream signaling. Our findings enrich the complicated network of EGFR signaling and uncover a molecular mechanism of FLCN in tumor suppression.

Increasing evidence has indicated that deletion of FLCN could lead to hyperactivation of various kinases, including ERK1/2 and AKT (Baba et al., 2008; Hasumi et al., 2009). Consistently, we demonstrate here that FLCN-deficiency reduces the rate of EGF-induced EGFR degradation, resulting in the continuous activation of ERK1/2 and AKT in an EGFR-dependent manner. Although biochemical analysis showed that the DENN domain, which is located in the C-terminal of FLCN, possesses GEF activity toward Rab35 (Nookala et al., 2012), activation of RAB35 by FLCN has never been validated in cells. Our results show that in HeLa cells, FLCN binds to Rab35 by its C-terminal, which plays a key role in mediating EGF-induced Rab35 activation and EGFR degradation.

Rab35 has been shown to regulate cargo recycling at endosomes, with an essential role in the regulation of actin cytoskeleton organization (Zhang et al., 2009; Chaineau et al., 2013). It is reported that Rab35 suppresses cell migration by controlling intracellular trafficking and cell surface levels of cadherins and β1-integrin in mammalian cells (Allaire et al., 2013; Charrasse et al., 2013; Argenzio et al., 2014; Tang et al., 2015). Consistent with these studies, our results show that Rab35 increases the rate of EGF-induced EGFR degradation and attenuates its downstream signaling. Furthermore, FLCN functions as an activator toward Rab35 in the process of EGF-stimulation. However, a recent report revealed that depletion of Rab35 inhibits AKT activation in response to growth factors through an underlying PI3K-dependent manner (Wheeler et al., 2015). The conflicting effect of Rab35 activation on AKT activity might depend on the cell context or experimental conditions. We previously demonstrated that Rab35 mediates Wnt5a-induced cell migration and EGF induces epithelial-mesenchymal transition via decreasing Wnt5a transcription (Zhu et al., 2013; Zhang et al., 2015). FLCN is also associated with Wnt signaling (Luijten et al., 2013). Our current observations, together with these previous studies, suggest that FLCN-Rab35 may play an essential role in the cross-talk between EGFR and Wnt signaling, which is necessary for further studies to elucidate the bilateral function of FLCN and Rab35 during oncogenic transformation.

When EGF-induced activation of EGFR signaling is completely blocked, EGFR trafficking is also obstructed in the cells pretreated with erlotinib. This suggests that the regulators of EGFR trafficking may sense EGFR signaling. Determining the upstream regulation mechanism of proteins associated with EGFR trafficking in detail will contribute to the comprehensive understanding of EGFR signal network. In summary, the finding that FLCN/Rab35 pathway contributes to the degradation of EGFR and attenuation of its downstream signaling advances our understanding of EGFR trafficking. However, it remains unclear whether FLCN and Rab35 act by binding to EGFR directly or via an indirect mechanism, further studies are required to investigate how the FLCN/Rab35 pathway promotes EGFR degradation and the association between EGFR and FLCN in clinical samples.

## Author contributions

JZ designed the study. JZ, SS, JC, and YZ performed the experiments. JZ performed the statistical analysis. JD and BD drafted the manuscript, YZ supervised the experimental work. All authors read and approved the final manuscript.

### Conflict of interest statement

The authors declare that the research was conducted in the absence of any commercial or financial relationships that could be construed as a potential conflict of interest.

## References

[B1] Allaire P. D. Seyed Sadr M. Chaineau M. Seyed Sadr E. Konefal S. Fotouhi M.. (2013). Interplay between Rab35 and Arf6 controls cargo recycling to coordinate cell adhesion and migration. J. Cell Sci. 126(Pt 3), 722–731. 10.1242/jcs.112375 23264734

[B2] Argenzio E. Margadant C. Leyton-Puig D. Janssen H. Jalink K. Sonnenberg A.. (2014). CLIC4 regulates cell adhesion and beta1 integrin trafficking. J. Cell Sci. 127, 5189–5203. 10.1242/jcs.150623 25344254

[B3] Avraham R. Yarden Y. (2011). Feedback regulation of EGFR signalling: decision making by early and delayed loops. Nat. Rev. Mol. Cell Biol. 12, 104–117. 10.1038/nrm3048 21252999

[B4] Baba M. Furihata M. Hong S. B. Tessarollo L. Haines D. C. Southon E.. (2008). Kidney-targeted Birt-Hogg-Dube gene inactivation in a mouse model: Erk1/2 and Akt-mTOR activation, cell hyperproliferation, and polycystic kidneys. J. Natl. Cancer Inst. 100, 140–154. 10.1093/jnci/djm288 18182616 PMC2704336

[B5] Chaineau M. Ioannou M. S. McPherson P. S. (2013). Rab35: GEFs, GAPs and effectors. Traffic 14, 1109–1117. 10.1111/tra.12096 23905989

[B6] Charrasse S. Comunale F. De Rossi S. Echard A. Gauthier-Rouviere C. (2013). Rab35 regulates cadherin-mediated adherens junction formation and myoblast fusion. Mol. Biol. Cell 24, 234–245. 10.1091/mbc.E12-02-0167 23197472 PMC3564529

[B7] Deng W. Wang Y. Gu L. Duan B. Cui J. Zhang Y.. (2016). MICAL1 controls cell invasive phenotype via regulating oxidative stress in breast cancer cells. BMC Cancer 16:489. 10.1186/s12885-016-2553-1 27430308 PMC4950114

[B8] Deribe Y. L. Wild P. Chandrashaker A. Curak J. Schmidt M. H. Kalaidzidis Y.. (2009). Regulation of epidermal growth factor receptor trafficking by lysine deacetylase HDAC6. Sci. Signal. 2:ra84. 10.1126/scisignal.2000576 20029029

[B9] Duan B. Cui J. Sun S. Zheng J. Zhang Y. Ye B.. (2016). EGF-stimulated activation of Rab35 regulates RUSC2-GIT2 complex formation to stabilize GIT2 during directional lung cancer cell migration. Cancer Lett. 379, 70–83. 10.1016/j.canlet.2016.05.027 27238570

[B10] Er E. E. Mendoza M. C. MacKey A. M. Rameh L. E. Blenis J. (2013). AKT facilitates EGFR trafficking and degradation by phosphorylating and activating PIKfyve. Sci. Signal. 6:ra45. 10.1126/scisignal.2004015 23757022 PMC4041878

[B11] Fujii H. Jiang W. Matsumoto T. Miyai K. Sashara K. Ohtsuji N.. (2006). Birt-Hogg-Dube gene mutations in human endometrial carcinomas with microsatellite instability. J. Pathol. 209, 328–335. 10.1002/path.1992 16691634

[B12] Goncharova E. A. Goncharov D. A. James M. L. Atochina-Vasserman E. N. Stepanova V. Hong S. B.. (2014). Folliculin controls lung alveolar enlargement and epithelial cell survival through E-cadherin, LKB1, and AMPK. Cell Rep. 7, 412–423. 10.1016/j.celrep.2014.03.025 24726356 PMC4034569

[B13] Goueli B. S. Powell M. B. Finger E. C. Pfeffer S. R. (2012). TBC1D16 is a Rab4A GTPase activating protein that regulates receptor recycling and EGF receptor signaling. Proc. Natl. Acad. Sci. U.S.A. 109, 15787–15792. 10.1073/pnas.1204540109 23019362 PMC3465424

[B14] Guda K. Veigl M. L. Varadan V. Nosrati A. Ravi L. Lutterbaugh J.. (2015). Novel recurrently mutated genes in African American colon cancers. Proc. Natl. Acad. Sci. U.S.A. 112, 1149–1154. 10.1073/pnas.1417064112 25583493 PMC4313860

[B15] Gyorffy B. Lanczky A. Eklund A. C. Denkert C. Budczies J. Li Q.. (2010). An online survival analysis tool to rapidly assess the effect of 22,277 genes on breast cancer prognosis using microarray data of 1,809 patients. Breast Cancer Res. Treat. 123, 725–731. 10.1007/s10549-009-0674-9 20020197

[B16] Hasumi Y. Baba M. Ajima R. Hasumi H. Valera V. A. Klein M. E.. (2009). Homozygous loss of BHD causes early embryonic lethality and kidney tumor development with activation of mTORC1 and mTORC2. Proc. Natl. Acad. Sci. U.S.A. 106, 18722–18727. 10.1073/pnas.0908853106 19850877 PMC2765925

[B17] Hunter T. (2000). Signaling–2000 and beyond. Cell 100, 113–127. 10.1016/S0092-8674(00)81688-8 10647936

[B18] Jiang W. Fujii H. Matsumoto T. Ohtsuji N. Tsurumaru M. Hino O. (2007). Birt-Hogg-Dube (BHD) gene mutations in human gastric cancer with high frequency microsatellite instability. Cancer Lett. 248, 103–111. 10.1016/j.canlet.2006.06.005 16870330

[B19] Jones S. Rappoport J. Z. (2014). Interdependent epidermal growth factor receptor signalling and trafficking. Int. J. Biochem. Cell Biol. 51:23–28. 10.1016/j.biocel.2014.03.014 24681003

[B20] Lee H. J. Zhuang G. Cao Y. Du P. Kim H. J. Settleman J. (2014). Drug resistance via feedback activation of Stat3 in oncogene-addicted cancer cells. Cancer Cell 26, 207–221. 10.1016/j.ccr.2014.05.019 25065853

[B21] Levi-Montalcini R. Cohen S. (1960). Effects of the extract of the mouse submaxillary salivary glands on the sympathetic system of mammals. Ann. N. Y. Acad. Sci. 85:324–341. 14416187 10.1111/j.1749-6632.1960.tb49963.x

[B22] Li J. Mansmann U. R. (2014). A molecular signaling map and its application. Cell. Signal. 26, 2834–2842. 10.1016/j.cellsig.2014.08.022 25192909

[B23] Luijten M. N. Basten S. G. Claessens T. Vernooij M. Scott C. L. Janssen R.. (2013). Birt-Hogg-Dube syndrome is a novel ciliopathy. Hum. Mol. Genet. 22, 4383–4397. 10.1093/hmg/ddt288 23784378 PMC3792695

[B24] Lynch T. J. Bell D. W. Sordella R. Gurubhagavatula S. Okimoto R. A. Brannigan B. W.. (2004). Activating mutations in the epidermal growth factor receptor underlying responsiveness of non-small-cell lung cancer to gefitinib. N. Engl. J. Med. 350, 2129–2139. 10.1056/NEJMoa040938 15118073

[B25] Medvetz D. A. Khabibullin D. Hariharan V. Ongusaha P. P. Goncharova E. A. Schlechter T.. (2012). Folliculin, the Product of the Birt-Hogg-Dube Tumor Suppressor Gene, Interacts with the Adherens Junction Protein p0071 to Regulate Cell-Cell Adhesion. PLoS ONE 7:e47842. 10.1371/journal.pone.0047842 23139756 PMC3490959

[B26] Nahorski M. S. Seabra L. Straatman-Iwanowska A. Wingenfeld A. Reiman A. Lu X.. (2012). Folliculin interacts with p0071 (plakophilin-4) and deficiency is associated with disordered RhoA signalling, epithelial polarization and cytokinesis. Hum. Mol. Genet. 21, 5268–5279. 10.1093/hmg/dds378 22965878 PMC3755511

[B27] Nickerson M. L. Warren M. B. Toro J. R. Matrosova V. Glenn G. Turner M. L.. (2002). Mutations in a novel gene lead to kidney tumors, lung wall defects, and benign tumors of the hair follicle in patients with the Birt-Hogg-Dube syndrome. Cancer Cell 2, 157–164. 10.1016/S1535-6108(02)00104-6 12204536

[B28] Nookala R. K. Langemeyer L. Pacitto A. Ochoa-Montano B. Donaldson J. C. Blaszczyk B. K.. (2012). Crystal structure of folliculin reveals a hidDENN function in genetically inherited renal cancer. Open Biol. 2, 120071. 10.1098/rsob.120071 22977732 PMC3438538

[B29] Patino-Lopez G. Dong X. Ben-Aissa K. Bernot K. M. Itoh T. Fukuda M.. (2008). Rab35 and its GAP EPI64C in T cells regulate receptor recycling and immunological synapse formation. J. Biol. Chem. 283, 18323–18330. 10.1074/jbc.M800056200 18450757 PMC2440627

[B30] Schlessinger J. (2000). Cell signaling by receptor tyrosine kinases. Cell 103, 211–225. 10.1016/S0092-8674(00)00114-8 11057895

[B31] Schmidt L. S. Linehan W. M. (2015). Molecular genetics and clinical features of Birt-Hogg-Dube syndrome. Nat. Rev. Urol. 12, 558–569. 10.1038/nrurol.2015.206 26334087 PMC5119524

[B32] Schmidt L. S. Nickerson M. L. Warren M. B. Glenn G. M. Toro J. R. Merino M. J.. (2005). Germline BHD-mutation spectrum and phenotype analysis of a large cohort of families with Birt-Hogg-Dube syndrome. Am. J. Hum. Genet. 76, 1023–1033. 10.1086/430842 15852235 PMC1196440

[B33] Sheach L. A. Adeney E. M. Kucukmetin A. Wilkinson S. J. Fisher A. D. Elattar A.. (2009). Androgen-related expression of G-proteins in ovarian cancer. Br. J. Cancer 101, 498–503. 10.1038/sj.bjc.6605153 19623182 PMC2720237

[B34] Sigismund S. Woelk T. Puri C. Maspero E. Tacchetti C. Transidico P.. (2005). Clathrin-independent endocytosis of ubiquitinated cargos. Proc. Natl. Acad. Sci. U.S.A. 102, 2760–2765. 10.1073/pnas.0409817102 15701692 PMC549482

[B35] Spangler J. B. Neil J. R. Abramovitch S. Yarden Y. White F. M. Lauffenburger D. A.. (2010). Combination antibody treatment down-regulates epidermal growth factor receptor by inhibiting endosomal recycling. Proc. Natl. Acad. Sci. U.S.A. 107, 13252–13257. 10.1073/pnas.0913476107 20616078 PMC2922117

[B36] Stenmark H. (2009). Rab GTPases as coordinators of vesicle traffic. Nat. Rev. Mol. Cell Biol. 10, 513–525. 10.1038/nrm2728 19603039

[B37] Tang Y. Lin Y. Li C. Hu X. Liu Y. He M.. (2015). MicroRNA-720 promotes *in vitro* cell migration by targeting Rab35 expression in cervical cancer cells. Cell Biosci. 5:56. 10.1186/s13578-015-0047-5 26413265 PMC4583841

[B38] Tebbutt N. Pedersen M. W. Johns T. G. (2013). Targeting the ERBB family in cancer: couples therapy. Nat. Rev. Cancer 13, 663–673. 10.1038/nrc3559 23949426

[B39] Tomas A. Futter C. E. Eden E. R. (2014). EGF receptor trafficking: consequences for signaling and cancer. Trends Cell Biol. 24, 26–34. 10.1016/j.tcb.2013.11.002 24295852 PMC3884125

[B40] Ullrich A. Coussens L. Hayflick J. S. Dull T. J. Gray A. Tam A. W.. (1984). Human epidermal growth factor receptor cDNA sequence and aberrant expression of the amplified gene in A431 epidermoid carcinoma cells. Nature 309, 418–425. 10.1038/309418a0 6328312

[B41] Walker F. Abramowitz L. Benabderrahmane D. Duval X. Descatoire V. Henin D.. (2009). Growth factor receptor expression in anal squamous lesions: modifications associated with oncogenic human papillomavirus and human immunodeficiency virus. Hum. Pathol. 40, 1517–1527. 10.1016/j.humpath.2009.05.010 19716155

[B42] Wheeler D. B. Zoncu R. Root D. E. Sabatini D. M. Sawyers C. L. (2015). Identification of an oncogenic RAB protein. Science 350, 211–217. 10.1126/science.aaa4903 26338797 PMC4600465

[B43] Zhang J. Fonovic M. Suyama K. Bogyo M. Scott M. P. (2009). Rab35 controls actin bundling by recruiting fascin as an effector protein. Science 325, 1250–1254. 10.1126/science.1174921 19729655

[B44] Zhang Y. Du J. Zheng J. Liu J. Xu R. Shen T.. (2015). EGF-reduced Wnt5a transcription induces epithelial-mesenchymal transition via Arf6-ERK signaling in gastric cancer cells. Oncotarget 6, 7244–7261. 10.18632/oncotarget.3133 25779663 PMC4466682

[B45] Zhu Y. Shen T. Liu J. Zheng J. Zhang Y. Xu R.. (2013). Rab35 is required for Wnt5a/Dvl2-induced Rac1 activation and cell migration in MCF-7 breast cancer cells. Cell. Signal. 25, 1075–1085. 10.1016/j.cellsig.2013.01.015 23353182

